# Acute Inflammation around the Upper Cervical Vertebræ

**Published:** 1893-03

**Authors:** John Kent Spender

**Affiliations:** Physician to the Royal Mineral Water Hospital, Bath.


THE BRISTOL

Medico-Chirurgical Journal.

MARCH, 1893.

### ACUTE INFLAMMATION AROUND THE UPPER CERVICAL VERTEBRÆ.

### A SUBJECTIVE CLINICAL STUDY.

BY

JOHN KENT SPENDER, M.D. Lond.,

*Physician to the Royal Mineral Water Hospital, Bath.*

"Let me be sick myself, if sometimes the malady of my patient be not a disease unto me."—Sir THOMAS BROWNE, M.D.

THERE is an old and ragged piece of wit which tells doctors to take their own medicines. With a glance which is a note of scorn the physician is invited to partake of what he so freely and cheaply orders for others! Every medical man ought (so it has been said) to try a new drug upon himself before he tests its efficacy in a patient. I have done this often, but the experiment has seldom made me wiser or better. It has been, generally, so devoid of physiological passion and interest, that the simple dulness of the thing has deterred me from further trials.

The doctrine of medical self-sacrifice has risen to a much higher level. A transcendental view is that it is the *function* (in the language of mathematics) of a healer to suffer himself as well as to cure suffering in others. It is a mark of probationary obedience to *try diseases upon ourselves;* to lie in philosophic calm and watch the solemn order of our symptoms. Against his will and without knowing that he has run into danger, a physician may become deadly sick; he is suddenly in the midst of a severe battle, and cannot run away. Courage and even defiance he may show; but I recommend him a quiet submission to his destiny. Heart and nerves are more likely to stand the ordeal when the temper is in a sedative mood. Let us keep our emotional forces still. Say to the unwelcome guest—"Sit down and do your mischief as quickly as you can; dwell in my poor tabernacle your allotted time; but spare me the havoc and the pain, if such be the will of God."

The loftiest chord is struck by the great Norwich physician, whose words are quoted at the head of this paper. The man who is in diagnostic darkness about a case is invited to make himself ill in the same way; and when the illness comes, he is to watch its cruel journey through his own body. He looks into his system, counts the throbs and thrills, and studies the pains of phantasmal nerves. If this purifying chastisement be not enough, then he should approach near enough to death to understand its pangs and terrors. Will anyone try Sir Thomas Browne's plan? But if we faint and fail to reach such a romantic standard, we may at least acquiesce in more modest ideals. If there be any clinical school in which a doctor ought to live and learn, it is his own bed of sickness. Nowhere else can the grammar and syntax of Disease be so thoroughly mastered. Even Dr. Beale's "minor ailments" (some of them are anything but "minor") may be thus enjoyed in an impressive manner, and "learnt by heart" by the dullest intellect. A clinical vivisection on these academic lines strengthens the texture of personal character, and adds to our store of working knowledge.

The subject of my present paper is a sudden and serious illness which overtook me in the summer of 1891. The illness had, so to speak, only a physical side to it. I was away from home, and was, therefore, undistracted by the worry which comes from loss of patients and apathy of friends. Mind and conscience were not only tranquil, but buoyant with the prospect of a refreshing holiday. Innocent plans had been made, and among the charming things proposed to be done was a short stay at Dovedale, in Derbyshire; not for the predatory purpose of killing fish, but from the more benign desire of loitering about a bit of lovely scenery. In the neighbourhood of the dale stands a grand old manorial farm, metamorphosed so as to be now a comfortable hostel for strangers. It is dignified by the emblematic name of the Izaak Walton Hotel. Here my wife and I proposed to stay for a week or so—walking, driving, reading, playing chess, or any other recreative whim. For two or three days everything was sunshine. It did not seem necessary to apply to oneself the dark Shaksperian oracle that "to wilful men, The injuries that they themselves procure, Must be their schoolmasters." [1] For I was not consciously "wilful," nor did I know that I had brought "injuries" on myself by want of prudence or any other folly. And yet I was ignorantly walking by the side of a crater all the time.

In the days of giant clinical lectures, such as those delivered by Dr. Walshe during the middle of this century, a solemn and donnish feature was the recitation of the *Prodromata*. Every disease has its dawn and rise; and this twilight stage is comparable to the overture before an opera or an oratorio. Thus, every specific fever has its warning signs; and these are often so characteristic as to be a fixed element in the diagnosis. Now it is grand, even if not pleasant, to suffer a *prodroma;* and the warnings which came to me were plain and loud, if hardness of heart and dulness of perception had not overlooked them. (*a*) In May or June, 1891, a great number of minute yellow freckles appeared on both arms. This is a minatory clause in the creed of Rheumatism. (*b*) Quasi-syncopal attacks; sometimes breathlessness; an instinctive placing of the hand over the region of the heart as if to support it. [2] (*c*) Polyuria, and the specific gravity of the urine always below 1006. Rapid decomposition of the secretion, which was very pale. Add to

[1] *King Lear*, II. iv. 305-7.

[2] I had nearly forgotten the persistent tachycardia, which began in April. Dr. E. Long Fox gives a vivid portraiture of the physiological riot which goes on in and around the heart when its rhythm is persistently disturbed. (*Influence of the Sympathetic on Disease*, pp. 74–6.)

these subjective points the canker and care of some long and anxious cases in practice; and the supreme insanity of letting nearly ten months pass without the rest and recreation of a single "day in the country."[1] During July there was sleeplessness; and the tongue was covered habitually with a blackish-yellow fur. Thus the organism had run down to a low level of daily health, and had become a fuel ripe and ready for any naughty germs that might fly in its way.

At last the day came (August 5th) for release. But that release should have taken a different form; and here was the initial blunder. Do not always say to a sore and weary man—"Go away, my dear fellow, as soon as you can; leave your work and run to the sea or the mountains." Facile and cheap advice, scarcely worth the proffered fee. More wise and homely is the counsel—"Stay in bed for two or three days; enjoy absolute quiet; rest and sleep, as you will; and take only simple fluid food." This has the note of common sense; but when does a doctor apply common sense to himself?[2] A silent time in bed means the immersion of the body in a physiological bath of warm air; the rate of metabolism is reduced to its lowest point; and if the nervous system be guarded from noise and worry, the equilibrium of function is soon restored.

We halted at Cheltenham for a few hours, and reached Dovedale on the evening of Thursday, August 6th. The next two days were spent in strolling about; but on Saturday afternoon, when coming out of the tortuous and beautiful enclosure called *par excellence* Dovedale, a cool damp wind blew

[1] On Sunday, June 14th, 1891, I rambled during a day of magnificent weather in the "Pleasure-grounds" of Stourton Park. At the entrance is an object which must be dear to Bristol people—the High Cross, erected in that city about 1373, as a mark of gratitude to Edward III. It is an elaborate piece of stonework, decorated with the statues of eight monarchs: King John, Henry III., Edward I., Edward III., Henry VI., Elizabeth, James I., and Charles I. The last four were added in 1633, when the cross was enlarged and painted and gilded. It was taken down in 1733, and shortly afterwards given by Dean Barton, whose brother was rector of Stourton, to Mr. Henry Hoare, who re-erected it where it now stands.

[2] Whenever a client about-to-be quotes Aristotle's words that it is "better to die than disobey the doctor" (*ἄμεινόν ἐστι τῷ ἀνθρωπῷ ἀποθνήσκειν, ἢ ἀπειθεῖν τῷ ἰατρῷ*), I expect to have a specially troublesome person to deal with.

upon us, heated with walking in a confined valley. I was well clothed, but there could be no doubt of the immediate effect of this wind on the nerves of the skin. "Chill" and weakness were felt almost directly. The skin became cold and dry, and there had been clearly a small shock to the nervous system.

During all that evening I staid in a warm bedroom—a room girt about with walls of Cyclopean thickness, and immured in the oldest part of the "Izaak Walton." I followed the rudimentary instincts of primitive people, and ordered a good fire, hot gruel, and a warm bed. And then a salutary dose of castor oil. Popular suffrage invests these means with sovereign power; and they are at least simple and innocent. And I was so childish as to imagine that they would cure me in the magic hours of night, and make me new and well in the morning.

Sunday, August 9th, was a day worthy of November in its unceasing rain and wind. It brought no health or cheer. A new symptom aroused curiosity—rigidity of the spine. I could hardly get out of bed because the trunk of the body was so inflexibly straight; and the neck could not be rotated to any appreciable degree. I walked just once downstairs and up again like a stiff inelastic poker: and the head and shoulders were thrown back in order to keep the whole body in stable equilibrium. I could not take up anything from a chair, or throw the head back when drinking water from a tumbler. There was no pain on pressing the spine anywhere; but the whole vertebral column was as immoveable as if clasped with an armour of splints. The vertebral pillar is made firm and upright by inorganic material; and this could not have become inflamed or otherwise diseased within a few hours. A little pathological meditation convinced me, therefore, that my trouble in this respect was purely an affair of muscular rigidity. But such pronounced rigidity must be a reflex response to a deep-seated irritation. What could it be? [1]

[1] There are points of delicate touch between medical and surgical diagnosis: and here is one. Mr. Edmund Owen says that the earliest and most trustworthy sign of disease of the spine in children is the "stiff and straight pose of the body." A healthy child can put his head between his knees, and the back assumes a complete convex sweep. Mr. Owen's paper, neat and precise in demonstration, was published in the *Medical Press and Circular*, May 30th, 1891.

Evening approached, and the prospect was dark and grim. A great sweating began; the pulse rose to 120; but in the absence of a clinical thermometer I could only roughly guess the body-heat. No doctor lived within six miles, and none but the commonest drugs could be procured at a grocer's shop in the next village. The ordeal of undressing and getting into bed had to be endured; and the longer it was put off, the harder would be the agony. The stress of mischief was concentrating in or near the upper cervical vertebræ; and within quite a few hours there had come a general swelling of the connective tissue at the back of the neck. As yet, there were no specific neural sympathies. But doing nothing did not mend the ache or the fever, or help the "lame dog over the stile" of getting into bed.

I recollect little now about this "stile" except that somehow my garments were dragged from me, and that two or three people carried me like a straight log of timber to a warm and firm mattress. Then there was the fine adjustment of little pillows all around the head and neck, to keep everything as immoveable as possible in the absence of sandbags. And now began a night of horrors. Hot javelins of pain shot up from the inside of the spinal marrow to the head; the breath had a volcanic burning as it passed through the mouth; the perspiration was profuse and sour—true notes of pyrexial rheumatism. And the misery was increased by the impossibility of voiding an accumulating quantity of urine. Soon after four o'clock things became so grave that a man was sent on horseback to fetch a doctor from Ashbourne, six miles distant;[1] and the messenger was told to bring an ounce of salicin from a druggist. I hear even now the trot of that merciful horse, as it left the stable yard; and its returning trot was welcome indeed. Forty-eight grains of salicin in plain water were taken immediately. At half-past seven (an hour afterwards) 48 grains were taken again. And at half-past eight a third dose likewise. Milk and soda water were sipped to quench the awful thirst.

Before nine o'clock the doctor kindly came. He found the

[1] I refrain from mentioning the names of the medical friends who saw me now and hereafter; but my obligations to them are more than I can express.

body-temperature just under 100 degrees; and my own feeling was that the three doses of salicin had modified the pungent heat of skin. And now if eight or ten leeches had been applied to the neck, it would have been a fine move. The symptoms were somewhat perplexing; and in an adjoining room my doctor ventured the cheerful opinion that I was in the "greatest danger." Such a diagnosis is moral rather than scientific; but better to me than the most exact judgment was the relief of pressing emergencies, and the attention which made me clinically more comfortable. "Go on with your salicin, and take sufficient fluid food."

By half-past three in the afternoon one ounce of salicin had been administered; exactly 48 grains every hour. This audacity of medication meant that the patient was in a fighting temper—a temper begotten by the feeling that possibly Death and Physic were in mortal combat: that the enemy must be met with sword in hand, our decisive artillery being brought up with march and drum. Hesitate or speculate, and the battle is lost before you know it. Timid friends ask — Would you prescribe an ounce of salicin to be taken within nine hours by anyone else, suffer how he might? Dare and do. Many of our failures in medical workmanship come from the lack of strenuous therapeutic will. Meningeal inflammation at the upper part of the spinal cord means speedy effusion and pressure on vital knots and points—pathways of fibres going hither and thither and commanding essential functions. The most magnificent dosage of salicin does no harm; it kills the distemper [1] without a scrap of physiological mischief.[2] I did not quarrel with a transient deafness. A bulletin issued at four o'clock in the afternoon would have been favourable. Its medical language would have said that the brain was clear; that there had been no motor or sensory disturbance; and that the swelling at the back of the neck was receding, with a slight increase in the vertebral movement. At intervals of two hours

[1] *Distemper* is a regular Queen Anne's word. It abounds in the epistolary and other literature of the 18th century.

[2] I need hardly say that salicylate of soda could not have been given in these large and frequent doses without peril of prostration and severe collapse.

four more doses of salicin were taken; and the evening closed with the appropriate and old-world discipline of five grains of calomel.

The night was turbulent, but the storm and stress were gradually yielding. A nurse of the old school of all kindness and no cleverness was staying in the house; and we thankfully engaged her to do minor service in the night. Her duty at daybreak was to administer a saline purgative; and by degrees one realised the humiliation (never before known) of being fed from a feeding-cup, of being dragged about while the "bed was made," and of being washed and brushed by a hired servant. I asked my conscience what sins had been committed that I should be so abased? But the advantage of a doctor being his own patient lies in this:—he pities, perhaps, his poor sick organism, but he knows that he is under the rule of therapeutic law. No one is "trying" this or that thing upon him, and making him the victim of interesting experiments in pharmacology.

This day and the next were occupied with the steady routine of 30 grains of salicin every third hour (five doses in the daytime); and raw eggs were added to the soda water and milk. Twice every day two cups of strong tea soothed the nervous system and maintained the action of the skin.

Early on Thursday morning, August 13th, pain in the head suddenly increased, mostly in the occipital and mastoid regions. Basal meningitis seemed the natural sequel of what had gone before. Streams of hot water from a sponge were poured over the scalp; and at seven o'clock calomel and castor oil were taken together, with a flood of warm gruel soon afterwards. My medical friend thought me very ill when he called at eleven o'clock; but we were surprised and pleased when the body temperature was found to be not higher than 99.5 degrees. He recommended the outward application of ice; and a lady in a country house near let me have any quantity from her private cellar. The uncomfortable and menacing symptoms passed away towards evening. During the greater part of the day the salicin was discontinued; and at night 50 grains of bromide of sodium were distinctly beneficial.

It seemed a reasonable theory of my illness that there had been for some months either a defective metabolism, or an imperfect excretion of metabolic products. We were encouraged to hope that a critical moment had passed, and that our appeals to the depurating glands of the body had not been in vain. The term *uratosis* was proposed by Sir William Roberts to designate the precipitation of crystalline urates in the tissues and fluids of the body; and a term is needed to express a filling of the lymph-spaces of muscle with another sort of waste, and a sluggish lymph-current all over the body. The practical point to remember is that when urine passes for weeks and months of an exceedingly low specific gravity, an apparently healthy man is not only next door to being ill, but next door to a malign rheumatic pyrexia. Instead of an arthritic explosion of the conventional type, fibrous coverings of viscera (or viscera themselves) may be stricken by the poor and polluted blood. What determines the force and course of the morbid projectile it is often impossible to say.

On Friday morning, August 14th, convalescence may be said to have begun, inasmuch as I was able to rise and perform some elementary offices of toilette. The effort was premature, and the bed was more grateful than ever in the afternoon; and an intense sleep of more than two hours' duration was a lecture on my imprudence. These long day-sleeps were a splendid economy, for the nights were almost sleepless terrors. How and why?

If an erring function in a corner of the body be thoroughly studied during one's own illness, the memory of it will remain for ever. Now deep down behind the atlas and axis, and making these bones the fulcrum of action, is a group of small muscles which pass up to the occiput, and are inserted therein. They are intimately concerned with those rotatory movements of which the axis is the pivot. These muscles are named the *complexus*, the *rectus posticus major* and *minor*, and the *obliquus*. The innervation of all comes from the first cervical nerve on each side. Every night, after sleep for an hour or two, acute pain began at the line of insertion of the muscular tendons, from the mastoid process inwards and backwards over the occipital curves towards the middle line. A narrow defined ridge of agony. My opinion was (and still is) that the acute phase of a few days before had left crumbs or webs of lymph which irritated the first cervical nerve, and provoked a muscular cramp. As the spasm was bilateral, the head was not inclined to either side; but the phenomenon was favoured by sleep and by the supine position.

In order to carry out properly those details of treatment which were imperatively required during the night, a trained nurse was summoned from a large institution at Stoke-on-Trent. The person who came was ideal in some points—thirty-five years old, strong, and ugly; but her skill was blended with a sharp temper, thus reversing the qualities of my former attendant. It was resolved that the regimen of the night (from ten p.m. to six a.m.) should be according to this pattern: (*a*) plenty of hot milk and broth; (*b*) moderate doses of salicin and bromide of sodium at intervals of three hours; (*c*) the free application of ice to the back of the neck and *points douloureux* of the scalp, whenever sleep was abruptly disturbed by pain. The day dietary was enriched and varied by fish, fowl, and vegetable soups. And a seidlitz powder was recommended on every second or third morning.

It suffices now to continue my chronicle in broad outline. The days became brighter and better in every respect; and although I could not read or think, chess and one or two letters were a regular morning recreation. But the nights were as one tossing in a wilderness, ending only with the gracious tea soon after six o'clock. On August 22nd, I walked through country lanes; on the 24th, we had a refreshing drive over hill and dale; and on the 25th, in wind and rain, we thankfully bade an everlasting good-bye to the Izaak Walton Hotel.

A few quiet days, with sun and breeze, were spent at Morthoe, near Ilfracombe.

On September 1st, home was gladly seen again. To the learned professions, Home is a bureau of work and a school of industry, and represents as many burdens as joys. A few days of very moderate medical doings were disastrous in a high degree. Besides a feeling of unspeakable weakness, new symptoms developed. (*a*) The teeth became loose and insensitive, as if the mouth were lined with wooden pegs, without organic cohesion; the gums and cheeks were normal. (*b*) A dense yellow fur covered the whole tongue. (*c*) Yellow streaks appeared on the skin below the eyelids. (*d*) A dull leaden tint, beginning on the scalp, gradually spread to the forehead and temples. Was this due to backward pressure in the radicles of the frontal and facial veins; and this, in its turn, to a sluggish stream in the occipital vein and other small tributaries of the pterygoid plexus? (*e*) There were curious and droll mental delusions; such as a fixed belief (unshaken by argument) that two and two make five.

The last act of every drama ought to have a cheerful note; and my medical story shall not lack its tune. With the concurrence of a kind neighbour and friend, an expert neurologist came from London to see me on September 22nd; and we had what Bath doctors of the 18th century called a "complete consultation." What was my condition and fate? We had to take into account a long and painful illness in 1887, a peripheral neuritis of many spinal nerves. Every point was scrutinised; abstruse reflexes were unburied and made to speak; physiological centres and systems were tried and searched through. The diagnosis was clear and bright, and the prognosis quite exhilarating. My learned advisers had faith in medicines, and did not chant those mocking heroics which are the refuge of despair—"Go away for two months and do nothing." A healing plan of campaign was opened immediately. It was ordered that I should take a quarter of a grain of calomel every night for two or three weeks; moderate doses of salicylate of sodium and bromide of sodium in combination twice a day; and a sufficient quantity of effervescing sulphate of magnesium every third morning. And of course there was a code of sensible rules about diet.

Never was there a scholar more obedient to magisterial precepts. The poet's "loving discipline of pain" had been borne until it had become a fatigue and a moral irritation.[1] Everything was done, and every prophecy was fulfilled. Time

[1] Study the "Problem of Pain" in *Lux Mundi*, 12th Ed., 1891, p. 82.

was in my favour; the body was unpoisoned by what Milton calls "misused wine," and (it may be added) misused tobacco; and with the exception of one bad time in 1887, there was no record of any illness in my life since a tragic bit of scarlet fever in early childhood, exactly fifty years before. So that there was no personal merit in getting well; but the pilots were not the less to be praised for their skilful guidance of the ship.

There is a broad economic difference between the illness of a doctor and that of a layman. The laity like the sentiment of being a little "poorly;" human sympathies are stirred, and affectionate enquiries crowd the air. To be always "quite well, thank you," sounds as if one's texture were almost barbaric in its strength. Tell a client—"How robust and rosy you look—how strong you are!" and you offend because a certain delicacy of constitution is among the latest fashions. But if you ask a question about the state of the mind as if *that* were the infirmity, what angry surprise there would be! Sir James Paget wishes that there was as much ambition for really good health as there is for athletic strength or bravery. There is too much of the very opposite, and one hears people boast that they cannot do this or that; and they write or talk as if all others ought to be like them. It is as if a cripple should insist that others ought to limp as he does.[1]

But however interesting as a person an invalid doctor may be, the lay public think first of themselves; and their good wishes are conveyed in the inconvenient form of withholding confidence and support. Kind letters and messages reach the doctor's chamber, but in a professional sense they are barren. Gushing emotion does not clothe or feed, and its sterility is in exact proportion to its bulk. Wherefore I exhort my younger brethren—take care of your health; and never omit the lawful recreation of a yearly holiday. Take with you books of poetry and travel. The 18th-century Lord Chesterfield said that he never "killed his own meat;" and I recommend a higher aim than a sojourn in the same tract of country every autumn for the sake of slaying or maiming harmless fishes and birds. Mountains and glaciers may be too far; but lakes and hills and

[1] *Studies of old Case-books*, p. 166.

seas are within reach of the most frugal. Visit without ceasing fresh places at home and abroad; and in the course of time you will be able to speak of many spas and health-resorts with the authority which comes from personal acquaintance. And a most useful sort of knowledge I have found this to be.

About three months ago I saw in a medical journal the obituary notice of a physician who was very nearly my contemporary at King's College. He was eccentric as a student, and it was easy to foretell the centrifugal curves of the developed practitioner. Utterly sceptical about therapeutic realities, he was compelled by his position as assistant-physician to the Hospital to prescribe drugs for the out-patients. But when he knew that he was going to die, he applied to himself his own cold creed of negation. Sternly he refused all medicines, even those which would have relieved pain and afforded an euthanasia. Now the Shaksperian dirge may be true that "Physic must come to dust" no less than "Learning and the Sceptre."[1] But is it seemly for a physician himself to murder Medicine, and to triumph over its "dust" and ashes? On a famous Greek coin is a representation of Apollo in a chariot, discharging his arrows and laying low the pestilence as he slew the Python. If we have not the mythological precision of Apollo, we are bound by medical honour to do what we can. The history of my illness is a convincing attestation of the power of medicines. It has increased my strong faith in the scientific verities of our art; it would have made me believe if I had doubted before. Our *Codex* is a documentary testament of the experience of our forefathers. Pharmacopœia follows Pharmacopœia in apostolic succession; they are the authoritative voices of learned therapeutists; and our shame is great if we neglect or decline to use the treasures at our very feet.

[1] *Cymbeline*, IV. ii. 268-9.

